# Usefulness and safety of transbronchial lung cryobiopsy for reassessment of treatment in the clinical course of diffuse parenchymal lung disease

**DOI:** 10.1186/s12890-022-01838-x

**Published:** 2022-01-27

**Authors:** Yozo Sato, Tomohisa Baba, Hideya Kitamura, Takashi Niwa, Shigeru Komatsu, Eri Hagiwara, Tae Iwasawa, Koji Okudela, Tamiko Takemura, Takashi Ogura

**Affiliations:** 1 Department of Respiratory Medicine, Kanagawa Cardiovascular and Respiratory Center, 6-16-1 Tomiokahigashi, Kanazawa-ku, Yokohama-City, Kanagawa, 236-0051 Japan; 2 Department of Radiology, Kanagawa Cardiovascular and Respiratory Center, 6-16-1 Tomiokahigashi, Kanazawa-ku, Yokohama-City, Kanagawa, Japan; 3 Department of Pathology, Yokohama City University Graduate School of Medicine, 3-9 Fukuura, Kanazawa-ku, Yokohama-City, Kanagawa, Japan; 4 Department of Pathology, Japanese Red Cross Medical Center, 4-1-22 Hiroo, Shibuya-ku, Tokyo, Japan

**Keywords:** Transbronchial lung cryobiopsy, Diffuse parenchymal lung disease, Progressive fibrosing interstitial lung diseases, Reassessment

## Abstract

**Background:**

The usefulness and safety of transbronchial lung cryobiopsy (TBLC) for reassessment of diffuse parenchymal lung disease (DPLD) with progression is still unknown. Our purpose was to clarify the usefulness and safety of TBLC for reassessment of DPLD with progression.

**Methods:**

This retrospective study included 31 patients with DPLD diagnosed by surgical lung biopsy who progressed in the clinical course and underwent TBLC for reassessment between January 2017 and September 2019 at Kanagawa Cardiovascular & Respiratory Center. Two pulmonologists independently selected the clinical diagnosis, treatment strategy, and confidence level of the treatment strategy based on clinical and radiological information with and without pathological information from TBLC. A consensus was reached among the pulmonologists regarding the clinical diagnosis, treatment strategy, and confidence level of the treatment strategy. Complications of TBLC were also examined.

**Results:**

Seven (22.6%), 5 (16.1%), and 6 (19.4%) of clinical diagnosis was changed after TBLC for Pulmonologist A, for Pulmonologist B, and for consensus, respectively. The treatment strategy was changed in 7 (22.6%), 8 (25.9%), and 6 (19.4%) cases after TBLC for Pulmonologist A, for Pulmonologist B and for consensus, respectively. The definite or high confidence level of the consensus treatment strategy was 54.8% (17/31) without TBLC and 83.9% (26/31) with TBLC. There were 6 cases of moderate bleeding, but no other complications were noted.

**Conclusions:**

Pathological information from TBLC may contribute to decision-making in treatment strategies for the progression of DPLD, and it may be safely performed.

**Supplementary Information:**

The online version contains supplementary material available at 10.1186/s12890-022-01838-x.

## Background

Both idiopathic pulmonary fibrosis (IPF) and about 20–30% of non-IPF diffuse parenchymal lung diseases (DPLD) have a poor prognosis and develop a progressive fibrosis phenotype [1]. The INBUILD trial demonstrated the efficacy of nintedanib in patients with progressive fibrosing interstitial lung diseases (PF-ILD) [2]. Patients included in the INBUILD trial were defined as having progressed despite standard treatment with an agent for their respective disease. However, in real-world clinical practice, when disease progression occurs in DPLD under treatment, it is often difficult to determine whether the treatment is a sufficient standard treatment for each type of DPLD. In addition, reassessment of the diagnosis may sometimes be necessary, since the diagnosis might change over time [3].

The gold standard for the initial diagnosis of DPLD is surgical lung biopsy (SLB) [4]. However, SLB is not easily performed due to its risk at the time of reassessment for DPLD with progression. Recently, the usefulness of transbronchial lung cryobiopsy (TBLC), which is safer than SLB, has been recognized, and a high diagnostic concordance rate with SLB has been reported in the initial diagnosis of DPLD, including IPF [5–7]. Therefore, TBLC may be a good option for reassessment of DPLD in disease progression. However, there are no reports on the usefulness of TBLC as a reassessment of DPLD with progression. In addition, TBLC for DPLD with progression may have an increased risk of complications compared with TBLC at the initial diagnosis. We conducted a retrospective study to examine the usefulness and safety of TBLC for reassessment of DPLD with progression.

## Materials and methods

### Patients

We conducted a retrospective study of patients with DPLD diagnosed by SLB who progressed during the clinical course of the disease and underwent TBLC for reassessment between January 2017 and September 2019 at Kanagawa Cardiovascular & Respiratory Center. The indication for TBLC was determined by the attending physicians. Patients who underwent TBLC for the initial diagnosis of DPLD in the same period were also selected to compare the quality of specimens and safety with TBLC during the clinical course. This retrospective study was approved by the institutional review board of the Kanagawa Cardiovascular & Respiratory Center (KCRC-20-0029).

### Procedures

A flexible BF-1TQ290 bronchoscope (Olympus Corporation, Tokyo, Japan) and a 1.9-mm or 2.4-mm cryoprobe (Erbe Elektromedizin, Tübingen, Germany) were used in this procedure.

For TBLC, all patients were intubated with a flexible endotracheal tube under sedation with midazolam and fentanyl, and spontaneous breathing was maintained. A cryoprobe was inserted through the working channel of a flexible bronchoscope and placed into a subpleural location 1 cm away from the pleura under fluoroscopic guidance and activated for 6–7 s with the 1.9-mm probe or 4–5 s with the 2.4-mm probe. The frozen lung parenchyma, cryoprobe, and flexible bronchoscope were removed *en bloc*, and the samples were placed in formalin. A balloon catheter was placed at the entrance of the targeted bronchus and inflated immediately after the cryoprobe was removed. It was inflated until hemostasis was achieved [8]. Depending on oxygenation, sedation, and bleeding conditions, one to four specimens were obtained from different segments. Bronchoalveolar lavage was performed during the procedure. After checking for pneumothorax with chest X-ray, the patient was discharged the next day.

### Specimen evaluation

The quality of tissue specimens was classified into three groups: A, sufficient; B, insufficient; and C, not evaluable. The confidence level of the pathological diagnosis of the specimens was classified into three groups: A, definite; B, probable; and C, diagnosis only [9].

### Study design

First, two experienced pulmonologists independently selected a clinical diagnosis, treatment strategy, and confidence level of treatment strategy based on clinical and radiological information without pathological information from TBLC for each patient (including pathological information of SLB performed previously). Then, they selected a clinical diagnosis, treatment strategy, and confidence level of the treatment strategy by including pathological information from TBLC. We examined the concordance of treatment strategies between the two experienced pulmonologists. In addition, in cases of discrepancy, consensus clinical diagnosis, treatment strategy, and confidence level of treatment strategy were determined after discussion by both pulmonologists without pathological information from TBLC for each patient and then with pathological information. Changes in clinical diagnosis, treatment strategy, and confidence level of the treatment strategy after including pathological information from TBLC were examined. The treatment strategies were selected as follows: (1) introduction or intensification of corticosteroids and immunosuppressants, (2) introduction or modification of antifibrotic drugs, (3) antigen avoidance, and (4) follow-up or continuation of current treatment. The confidence level of the treatment strategy was defined as the percentage of confidence in the chosen treatment and was selected from definite (≥ 90%), high (70–90%), low (51–70%), and indeterminate (< 50%). The ontological theory of the diagnostic confidence level in ILD has been reported in past papers and is used in clinical practice [10]. We applied this to the confidence level of the treatment strategy.

### Complications

We examined the complications of TBLC, including pneumothorax, hemorrhage, and acute exacerbation. Severe bleeding was defined as the occurrence of hemodynamic or respiratory instability or the requirement for transfusion, any surgical intervention, or admission to the intensive care unit. Moderate bleeding was defined as the requirement of thrombin sprayed into the airway. In this study, we compared patients who underwent TBLC for the initial diagnosis of DPLD during the same period (between January 2017 and September 2019) on the incidence of complications of TBLC.

### Data analysis

Statistical analyses were performed using JMP 12 software (SAS Institute, Cary, NC). We used the chi-squared test for categorical variables and the t-test for continuous variables.

## Results

**Fig. 1:**
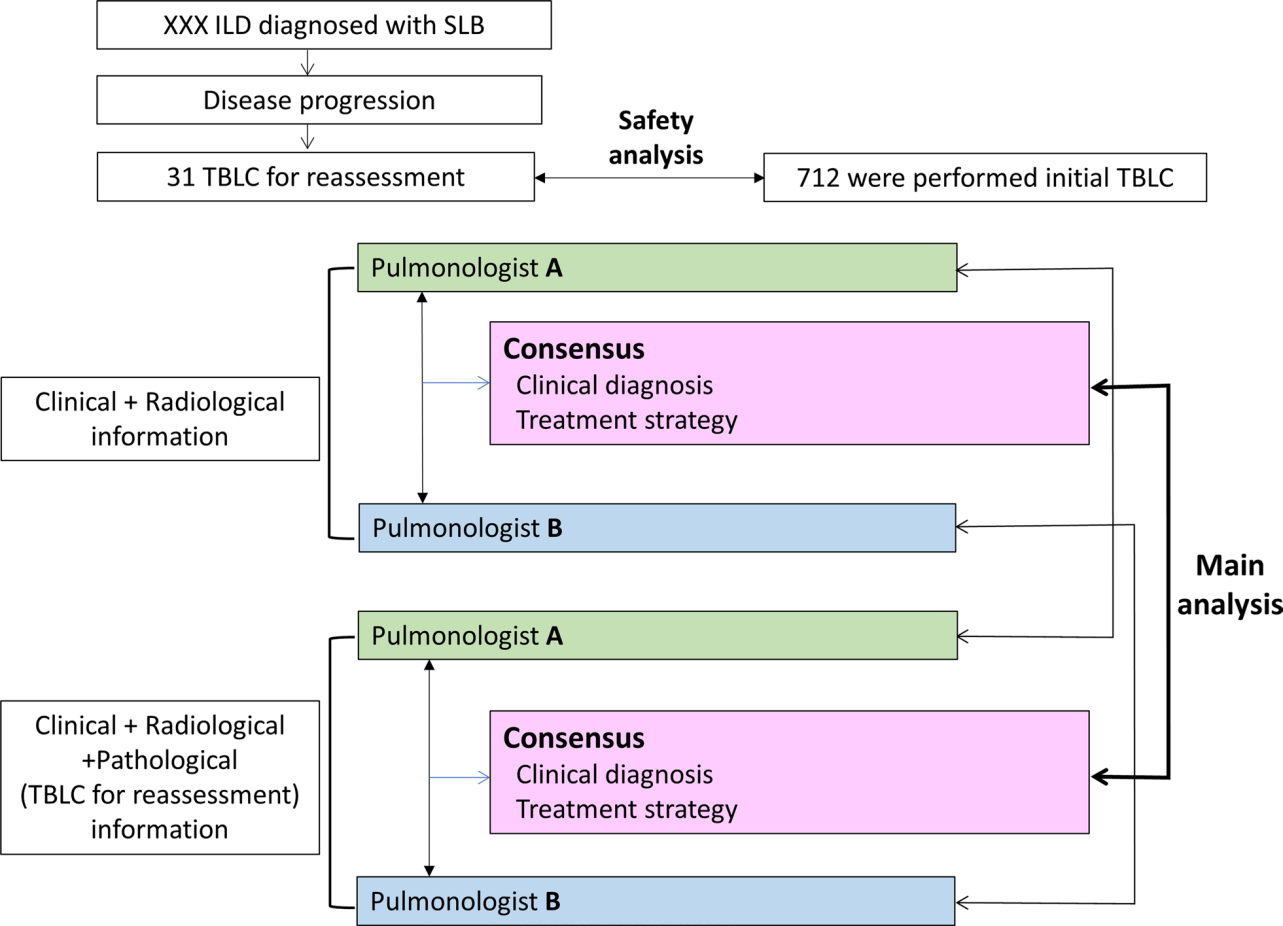
Study flowchart. TBLC was performed in 31 patients with diffuse parenchymal lung disease who underwent SLB followed by MDD and had disease progression. For each patient, clinical diagnosis, treatment strategy, and confidence level of treatment strategy based on clinical and radiological information with and without pathological information from TBLC were determined by Pulmonologist A, Pulmonologist B, and consensus between them. MDD, multi-disciplinary discussion; SLB, surgical lung biopsy; TBLC, transbronchial lung cryobiopsy

The study flowchart is shown in Fig. 1. This study included 31 patients, of whom 15 (48%) were women. The median age of patients at the time of TBLC was 71.5 years (range, 47–82 years) (Table 1). The diagnosis at SLB was 4 cases of IPF, 5 cases of idiopathic non-specific interstitial pneumonia (iNSIP), 10 cases of unclassifiable interstitial pneumonia (UCIP), 4 cases of connective tissue disease-associated interstitial lung disease (CTD-ILD), 4 cases of hypersensitivity pneumonia, and 4 cases of others. The chosen treatments at the time of TBLC included 12 cases of corticosteroids, 3 cases of immunosuppressants, 5 cases of antifibrotic drugs, and 16 cases of no medication. At the time of TBLC, 21 patients met the definition of PF-ILD in the INBUILD trial. The initial TBLC cases included 712 patients. Although the patients in this study were slightly older than the patients who underwent initial TBLC, there were no significant differences in sex, forced vital capacity (FVC), diffusing capacity for carbon monoxide (DLCO), number of specimens, quality of specimens, or pathological confidence level (Table 2).

**Table 1:** Baseline characteristics

Number of patients	31
Age (median, range)	71.5, 47–82
Gender(male/female)	16/15
Smoking history(never/ex)	16/15
6MWT	
Min SpO2(%)	88.1 ± 4.68
Total distance(m)	513 ± 84.0
Labo Data	
KL-6(U/ml)	1551 ± 1135
SP-D(ng/ml)	349 ± 222
Pulmonary function test	
%FVC(%)	86.4 ± 19.7
%DLCO(%)	77.7 ± 18.1
Diagnosis at SLB	
IPF	4
iNSIP	5
UCIP	10
CTD-ILD	4
HP	4
Others	4
Treatment	
Corticosteroids	12
Immunosuppressants	3(tacrolimus, cyclosporine, mycophenolate mofetil)
Antifibrotic drugs	5
No medication	16
LTOT	2
Period from SLB to TBLB(median, range)	49 months, 7–266 months

*6MWT* 6-Minute Walk Test, *KL-6* Sialylated carbohydrate antigen Krebs von den Lungen-6, *SP-D* pulmonary Surfactant Protein-D, *FVC* forced vital capacity, *DLCO* carbon monoxide diffusing capacity, *SLB* surgical lung biopsy, *IPF* idiopathic pulmonary fibrosis, *iNSIP* idiopathic nonspecific interstitial pneumonia, *UCIP* unclassifiable interstitial pneumonia, *CTD-ILD* connective tissue disease-interstitial lung disease, *HP* hypersensitivity pneumonia, *LTOT* long term oxygen therapy, *TBLB* transbronchial lung biopsy

**Table 2:** Baseline characteristics compared to initial TBLC

	reassessment TBLC	initial TBLC	P value
Number of patients	31	712	
Gender(male/female)	16/15	401/311	0.606
Age(median)	71.5	68	0.021
%FVC (%)	86.4	85.0	0.697
%DLCO (%)	77.7	74.6	0.468
Number of specimens 1/2/3/4	6/19/6/0	79/437/193/3	0.463
Quality score A/B/C	21/10/0	467/199/38	0.397
Pathological confidence A/B/C	11/19/1	281/339/83	0.215

*SLB* surgical lung biopsy, *TBLB* transbronchial lung biopsy, *FVC* forced vital capacity, *DLCO* diffusing capacity for carbon monoxide

**Fig. 2:**
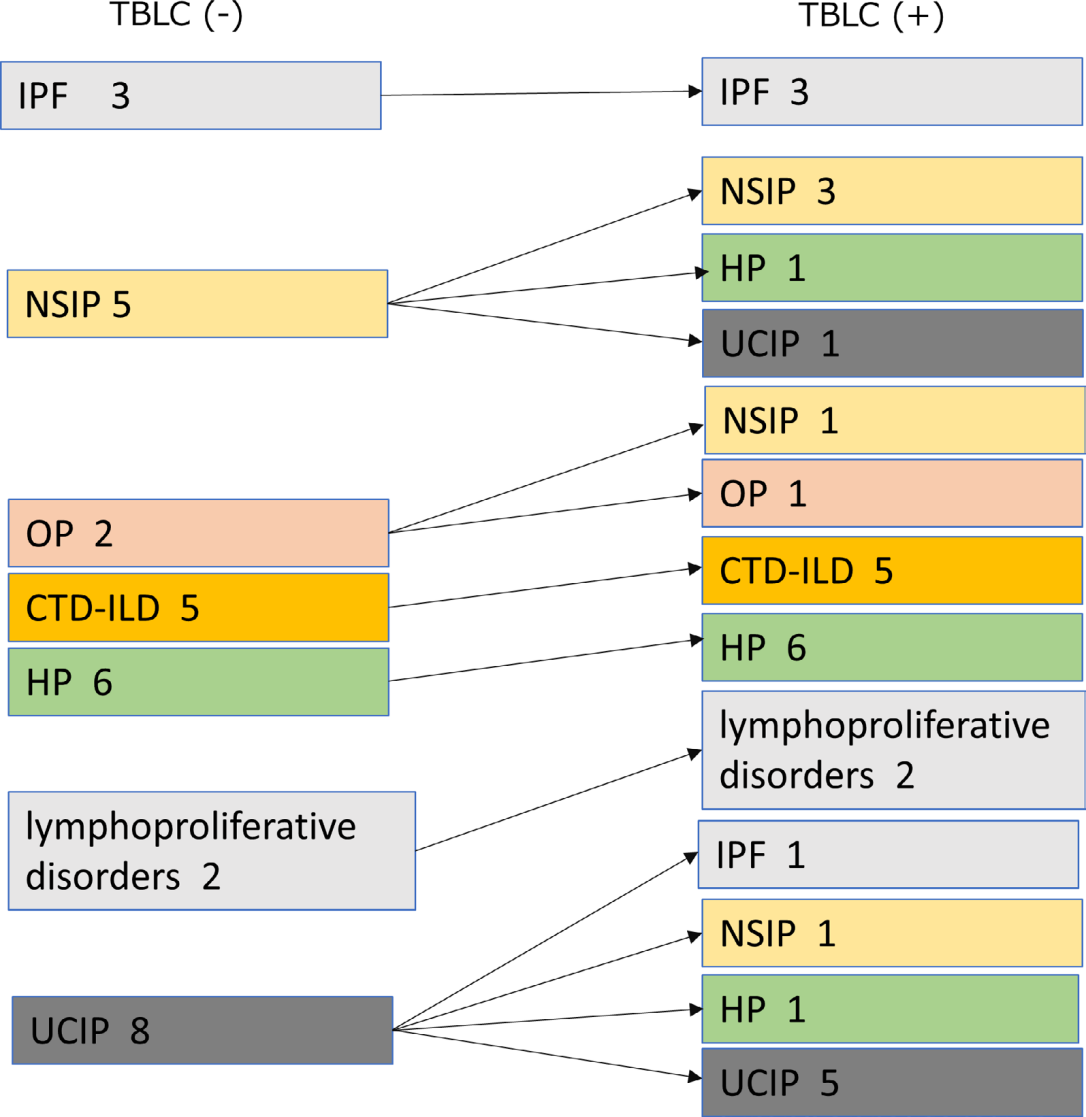
Clinical diagnosis of consensus of Pulmonologists A and B. Pulmonologists A and B reached a final consensus clinical diagnosis for each patient based on clinical and radiological information with or without pathological information from TBLC. There were 6 cases (19.4%) with a change in clinical diagnosis by TBLC. TBLC, transbronchial lung cryobiopsy; IPF, idiopathic pulmonary fibrosis; NSIP, idiopathic non-specific interstitial pneumonia; OP, organizing pneumonia; CTD-ILD, connective tissue disease-associated interstitial lung disease HP, hypersensitivity pneumonia; UCIP, unclassifiable interstitial pneumonia

**Fig. 3:**
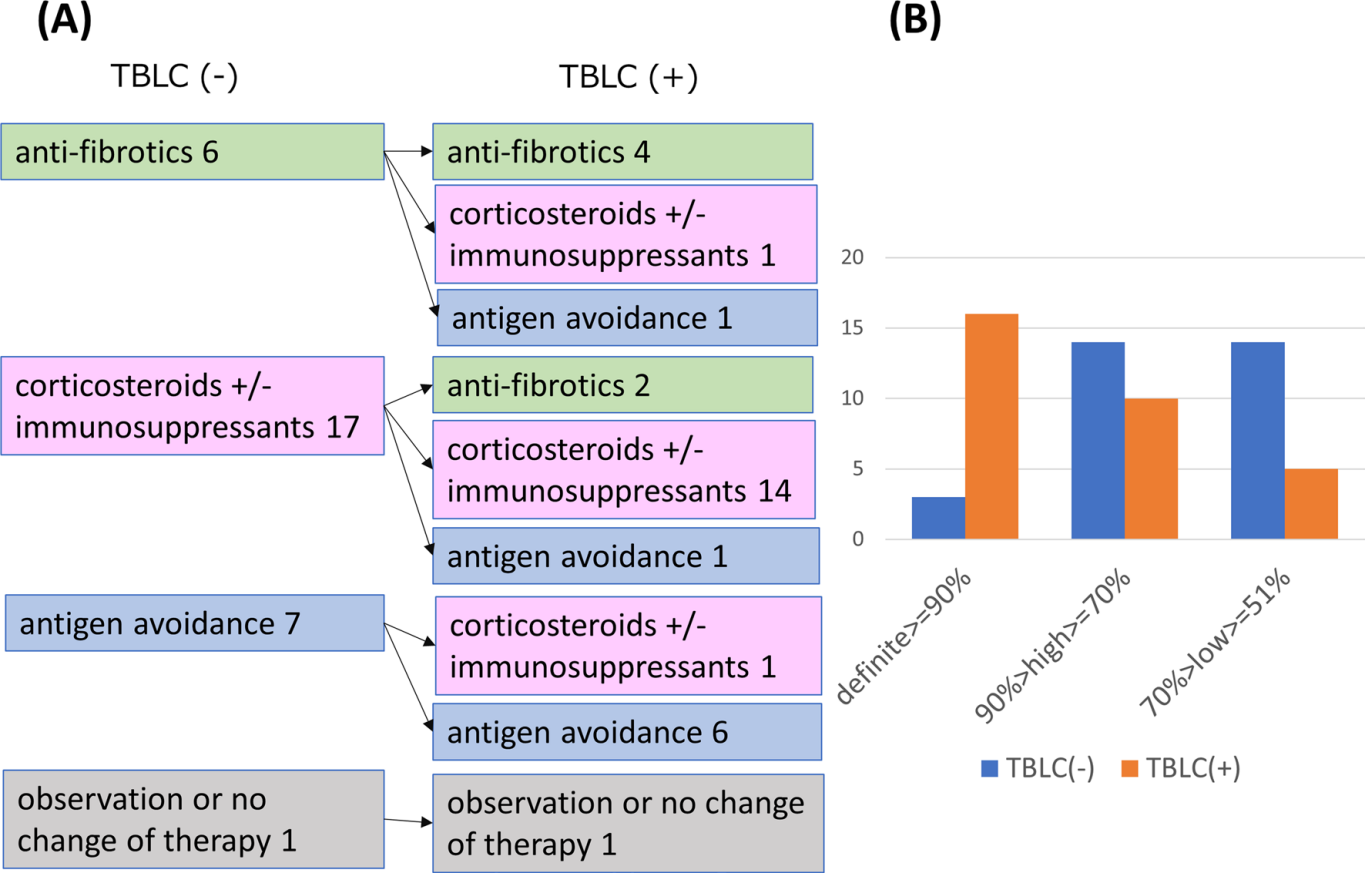
**A** Consensus treatment strategy. Pulmonologists A and B reached final consensus treatment strategies based on clinical and radiological information with or without pathological information from TBLC. There were 6 cases (19.4%) with a change in treatment strategy by TBLC. **B** Confidence level of consensus treatment strategy. Pulmonologists A and B reached final consensus confidence levels of consensus treatment strategies based on clinical and radiological information with or without pathological information from TBLC. A definite or high confidence level of the consensus treatment strategy was noted in 54.8% (17/31) of cases without TBLC and 83.9% (26/31) of cases with TBLC. TBLC, transbronchial lung cryobiopsy

**Fig. 4:**
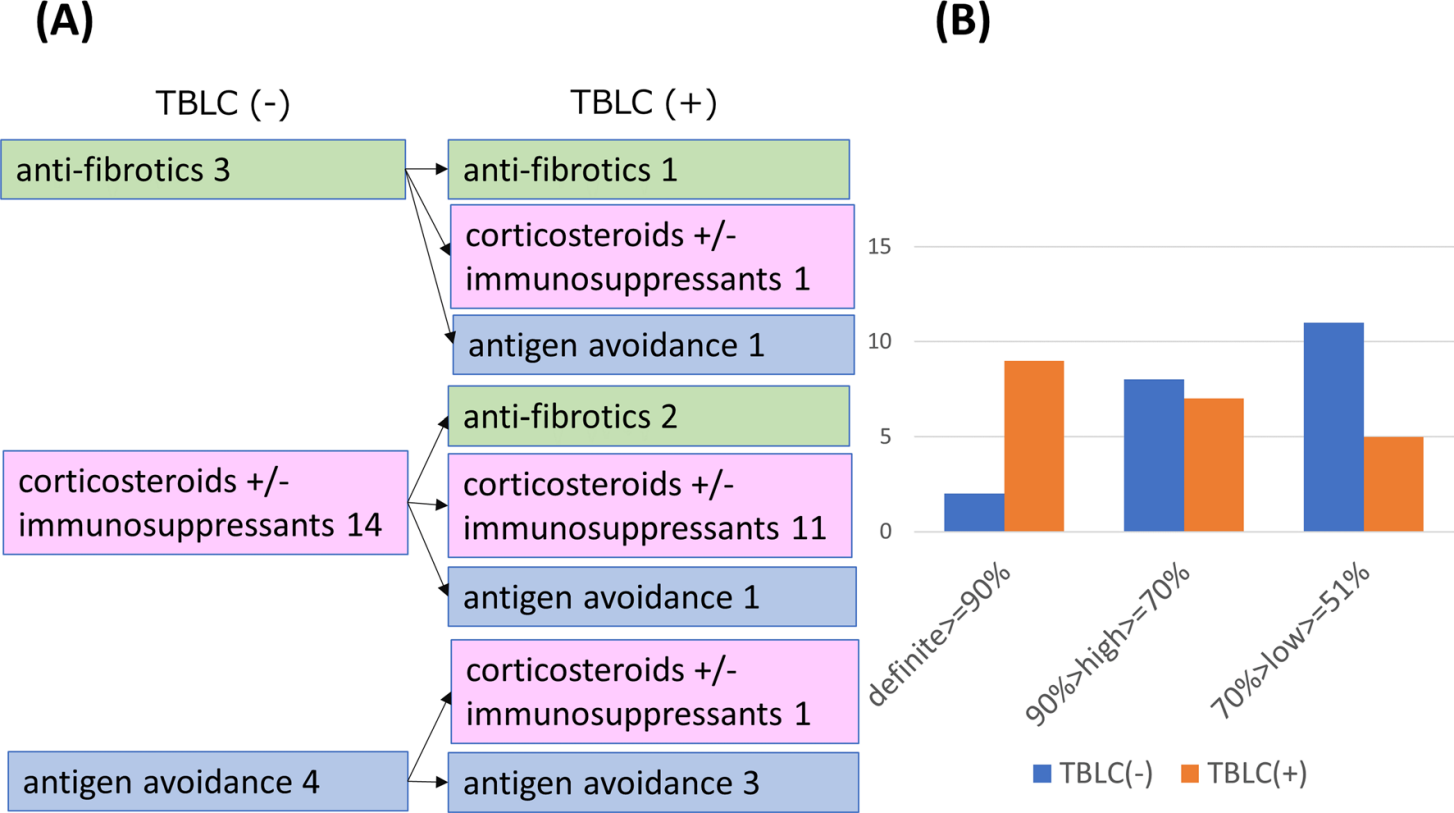
(**A**) Consensus treatment strategy in PF-ILD. Pulmonologists A and B reached final consensus treatment strategy based on clinical and radiological information without pathological information of TBLC and with pathological information of TBLC in PF-ILD patients. The change of treatment strategy by TBLC was 6/21 (28.6%). **B** Confidence level of consensus treatment strategy in PF-ILD. Pulmonologists A and B reached final confidence level of consensus treatment strategy based on clinical and radiological information without pathological information of TBLC and with pathological information of TBLC in PF-ILD patients. The definite or high confidence level of the consensus treatment strategy was 47.6% (10/21) without TBLC and 76.2% (16/21) with TBLC

The change in consensus clinical diagnosis (Fig. 2) and clinical diagnosis by each pulmonologist (Additional file 1: Fig. S1, Additional file 2: Fig. S2), consensus treatment strategy (Fig. 3A) and treatment strategy by each pulmonologist (Additional file 3: Fig. S3, Additional file 4: Fig. S4), and confidence level of treatment strategy (Fig. 3B) (Additional file 5: Fig. S5, Additional file 6: Fig. S6) with and without TBLC are summarized. Seven (22.6%), 5 (16.1%), and 6 (19.4%) of clinical diagnosis was changed after TBLC for Pulmonologist A, for Pulmonologist B, and for consensus, respectively. The treatment strategy was changed in 7 (22.6%), 8 (25.9%), and 6 (19.4%) cases after TBLC for Pulmonologist A, for Pulmonologist B and for consensus, respectively. The definite or high confidence level of the treatment strategy for Pulmonologist A was 51.6% (16/31) without TBLC and 83.9% (26/31) with TBLC. The definite or high confidence level of the treatment strategy for Pulmonologist B was 83.9% (26/31) without TBLC and 87.1% (27/31) with TBLC. The definite or high confidence level of the consensus treatment strategy was 54.8% (17/31) without TBLC and 83.9% (26/31) with TBLC. The concordance of treatment strategies between the two pulmonologists slightly increased by adding TBLC pathological information (without TBLC, agreement = 64.5%, κ = 0.47, 95% CI = 0.22–0.72; with TBLC, agreement = 64.5%, κ = 0.42, 95% CI = 0.14–0.69). Among 21 cases of PF-ILD, 6 (28.6%) cases had their treatment strategy changed after TBLC in consensus (Fig. 4A). A definite or high confidence level of the consensus treatment strategy was 47.6% (10/21) without TBLC and 76.2% (16/21) with TBLC (Fig. 4B).

There were no cases of pneumothorax, severe bleeding, or acute exacerbation but there were 6 cases of moderate bleeding (19.4%). There were no significant differences in the incidence of complications between the two groups (Table 3).

**Table 3:** Complications compared to initial TBLC

	Reassessment TBLC	Initial TBLC	*P* value
Number of patients	31	712	
Moderate–severe bleeding	19.4% (6)	17.1% (122)	0.749
Pneumothorax	0.0% (0)	6.3% (45)	0.149
Acute exacerbation	0.0% (0)	0.001% (1)	0.835

*TBLC* transbronchial lung cryobiopsy

## Discussion

The purpose of this study was to clarify the usefulness and safety of TBLC for reassessment when disease progression is observed during the course of diagnosis. TBLC for DPLD with progression resulted in a change in clinical diagnosis in about 20% of cases and a change in treatment strategy in about 20–25% of cases. The percentage of cases with a definite or high confidence level in the treatment strategy increased from about 50% to about 80% by adding TBLC. From these results, TBLC contributed to a change in clinical diagnosis and treatment strategy in some cases and increased the confidence level of the treatment strategy. Similarly, in the PF-ILD subgroup, TBLC contributed to a change in the treatment strategy and increased the confidence level of the treatment strategy.

The INBUILD trial showed that the annual rate of decline in FVC was significantly lower among patients who received nintedanib than among those who received placebo in patients with PF-ILD [2]. Thus, antifibrotic drugs in patients with PF-ILD have been shown to slow down disease progression. However, that study was a comparison with placebo, and it is possible that some PF-ILD cases may benefit more from immunosuppressive treatment. Patients included in the INBUILD trial were defined as having progressed despite standard treatment with an agent for their respective disease, but it is unclear whether standard treatment was adequate. The exclusion criteria in the INBUILD trial were patients who were treated with azathioprine, cyclosporine, mycophenolate mofetil, tacrolimus, rituximab, cyclophosphamide, or oral glucocorticoids (at a dose of > 20 mg per day for glucocorticoids). Because the patients did not receive the above treatment in the INBUILD trial, it is unclear whether adequate immunosuppressive treatment is being used in different types of DPLD. ILD is known to cause fibrosis by a common mechanism in late phases [11]. Although the usefulness of antifibrotic drugs in this phase has been demonstrated, it is possible that some PF-ILD cases may not have sufficiently suppressed inflammation. Clinical information makes it difficult to determine whether immunosuppressive treatment is adequate.

In this study, experienced pulmonologists selected to introduce or intensify corticosteroids and immunosuppressants in about half of the cases with or without pathological information from TBLC. These results suggest that some patients with PF-ILD may benefit from the introduction or intensification of immunosuppressive treatment rather than the use of antifibrotic drugs.

In DPLD, the early phase is disease-specific, and the disease causes chronic inflammation. Repeated alveolar or endothelial-cell injury or immune activation and inflammation is said to cause fibrosis [12]. Determining whether inflammation or fibrosis is the primary pathogenetic mechanism of disease progression may play an important role in selecting between antifibrotic drugs and immunosuppressive therapy. However, it is often difficult to assess by only clinical and radiological information as to whether inflammation or fibrosis is the primary mechanism of disease progression. In addition to clinical and radiological findings, pathological findings are important for determining the pathogenesis of the disease. Although SLB is a reliable method to evaluate pathological findings, it cannot be easily performed because of its risk, whereas TBLC is minimally risky and a good choice for evaluating pathological findings. In cases where the pathological findings show strong cellular infiltration, immunosuppressive therapy should be introduced or intensified, and in cases where there is little cellular infiltration and strong fibrosis, antifibrotic drugs may be a better choice. In this study, pathological information from TBLC allowed us to evaluate inflammatory cell infiltration and fibrosis, which may have contributed to the change in treatment strategy.

Complications, such as hemorrhage and pneumothorax, have been reported in TBLC. A systematic review by Ravaglia et al. reported 20.2% of patients experienced pneumothorax, 0.3% experienced acute exacerbations, and no patients experienced severe hemorrhage [5]. The incidence of complications such as pneumothorax and hemorrhage in this study was not higher than that of previous reports or data from our institution in initial TBLC, suggesting that TBLC can be safely performed even in patients with disease progression.

We acknowledge that there are several limitations to this study. First, this was a retrospective study with a small sample size. Second, the low rate of agreement among experienced respiratory pulmonologists means that the views of the pulmonologists in this study may be biased. Third, the clinical outcome could not be assessed because this was a retrospective study using only previously obtained samples.

## Conclusions

In conclusion, TBLC may be useful in reassessing treatment strategies in the clinical course of DPLD with progression, and the risk of complications may be similar to that of initial TBLC.

## Supplementary Information

**Additional file 1: Fig. S1A**. Clinical diagnosis of pulmonologist A. Pulmonologist A selected clinical diagnosis based on clinical and radiological information without pathological information of TBLC and with pathological information of TBLC. The change of clinical diagnosis by TBLC was 7/31 (22.6%).

**Additional file 2: Fig. S2**. Clinical diagnosis of pulmonologist B. Pulmonologist B selected clinical diagnosis based on clinical and radiological information without pathological information of TBLC and with pathological information of TBLC. The change of clinical diagnosis by TBLC was 5/31 (16.1%).

**Additional file 3: Fig. S3**. Treatment strategy of pulmonologist A. Pulmonologist A selected treatment strategy based on clinical and radiological information without pathological information of TBLC and with pathological information of TBLC. The change of treatment strategy by TBLC was 7/31 (22.6%).

**Additional file 4: Fig. S4**. Treatment strategy of pulmonologist B. Pulmonologist B selected treatment strategy based on clinical and radiological information without pathological information of TBLC and with pathological information of TBLC. The change of treatment strategy by TBLC was 8/31 (25.9%).

**Additional file 5: Fig. S5**. Confidence level of pulmonologist A treatment strategy. Pulmonologist A selected confidence level of treatment strategy based on clinical and radiological information without pathological information of TBLC and with pathological information of TBLC. The definite or high confidence level of treatment strategy was 51.6% (16/31) without TBLC and 83.9% (26/31) with TBLC.

**Additional file 6: Fig. S6**. Confidence level of pulmonologist B treatment strategy. Pulmonologist B selected confidence level of treatment strategy based on clinical and radiological information without pathological information of TBLC and with pathological information of TBLC. The definite or high confidence level of treatment strategy 83.9% (26/31) without TBLC and 87.1% (27/31) with TBLC.

## Data Availability

The dataset supporting the conclusions of this article is presented within the article. The detailed clinical data is not available because of patients’ confidentiality.

## Abbreviations

IPF: Idiopathic pulmonary fibrosis
DPLD: Diffuse parenchymal lung diseases
PF-ILD: Progressive fibrosing interstitial lung diseases
SLB: Surgical lung biopsy
TBLC: Transbronchial lung cryobiopsy
iNSIP: Idiopathic non-specific interstitial pneumonia
UCIP: Unclassifiable interstitial pneumonia
CTD-ILD: Connective tissue disease-associated interstitial lung disease
FVC: Forced vital capacity
DLCO: Diffusing capacity for carbon monoxide
