# Supplementary figures and images for: Usefulness and safety of transbronchial lung cryobiopsy for reassessment of treatment in the clinical course of diffuse parenchymal lung disease
Source: BMC Pulm Med. 2022 Jan 27;22:46. doi: 10.1186/s12890-022-01838-x (PMC8793241; doi:10.1186/s12890-022-01838-x)

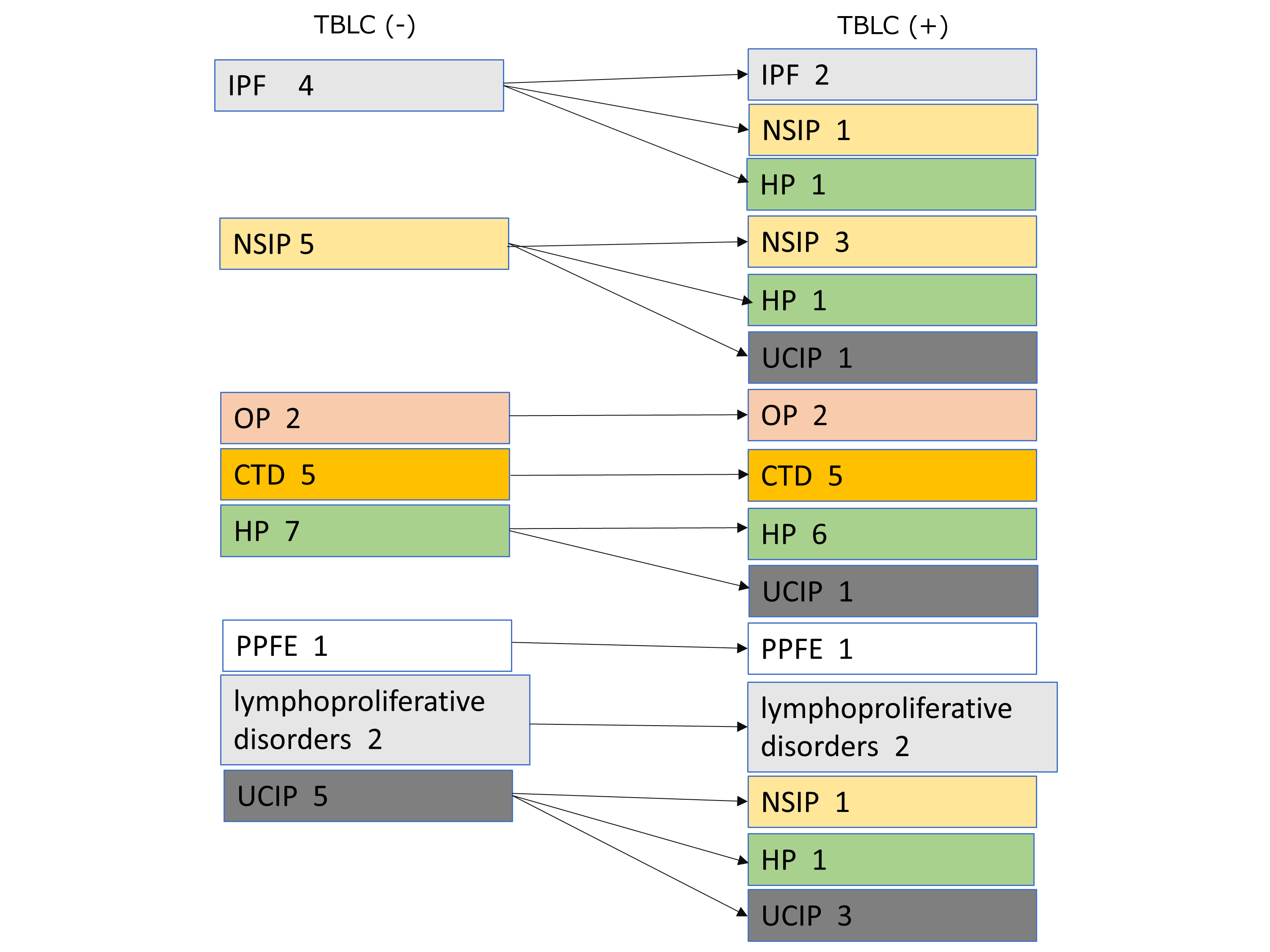

Supplement: Supplementary file 1 — Additional file 1: Fig. S1A. Clinical diagnosis of pulmonologist A. Pulmonologist A selected clinical diagnosis based on clinical and radiological information without pathological information of TBLC and with pathological information of TBLC. The change of clinical diagnosis by TBLC was 7/31 (22.6%). [file 12890_2022_1838_MOESM1_ESM.png]

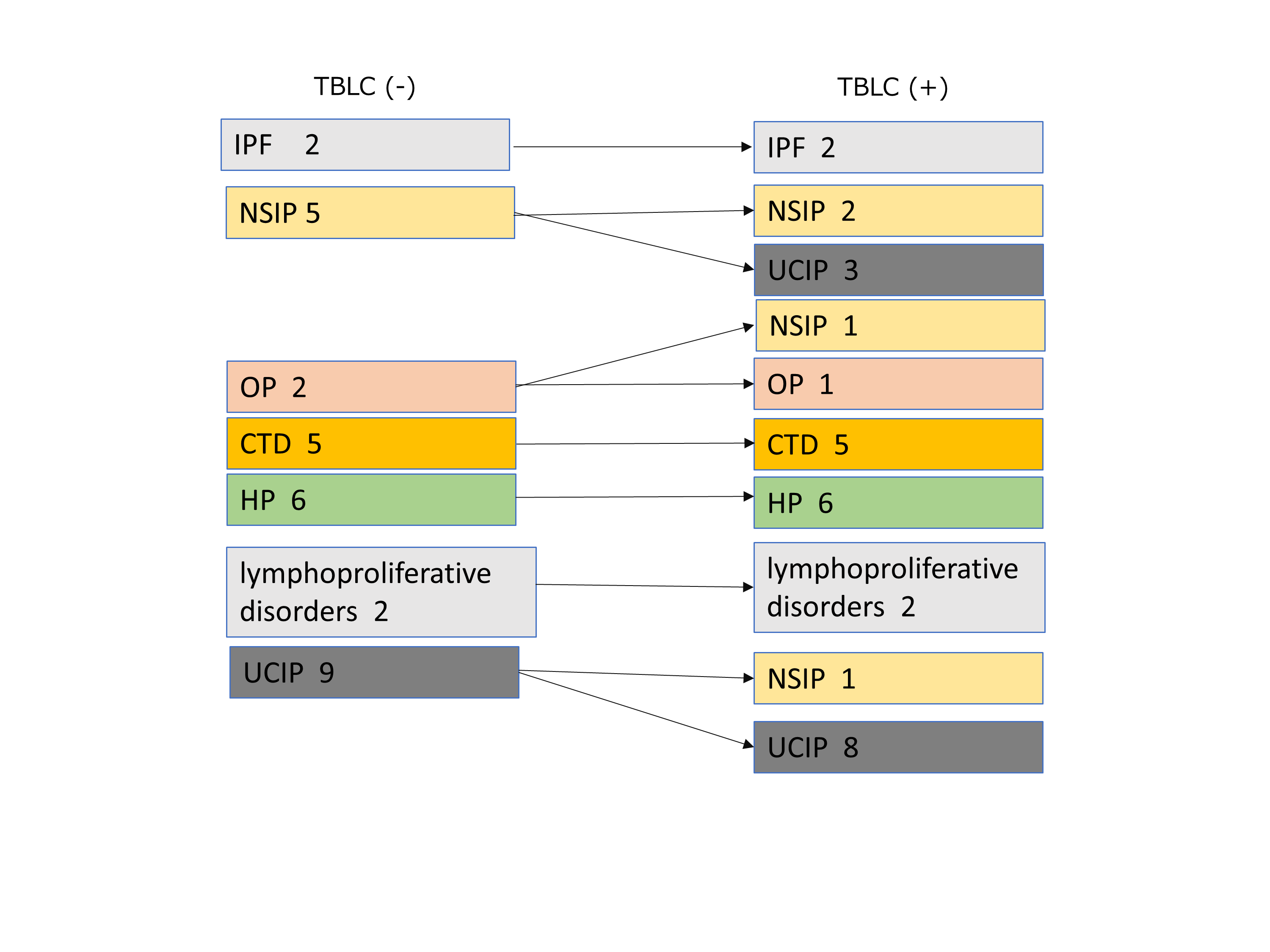

Supplement: Supplementary file 2 — Additional file 2: Fig. S2. Clinical diagnosis of pulmonologist B. Pulmonologist B selected clinical diagnosis based on clinical and radiological information without pathological information of TBLC and with pathological information of TBLC. The change of clinical diagnosis by TBLC was 5/31 (16.1%). [file 12890_2022_1838_MOESM2_ESM.png]

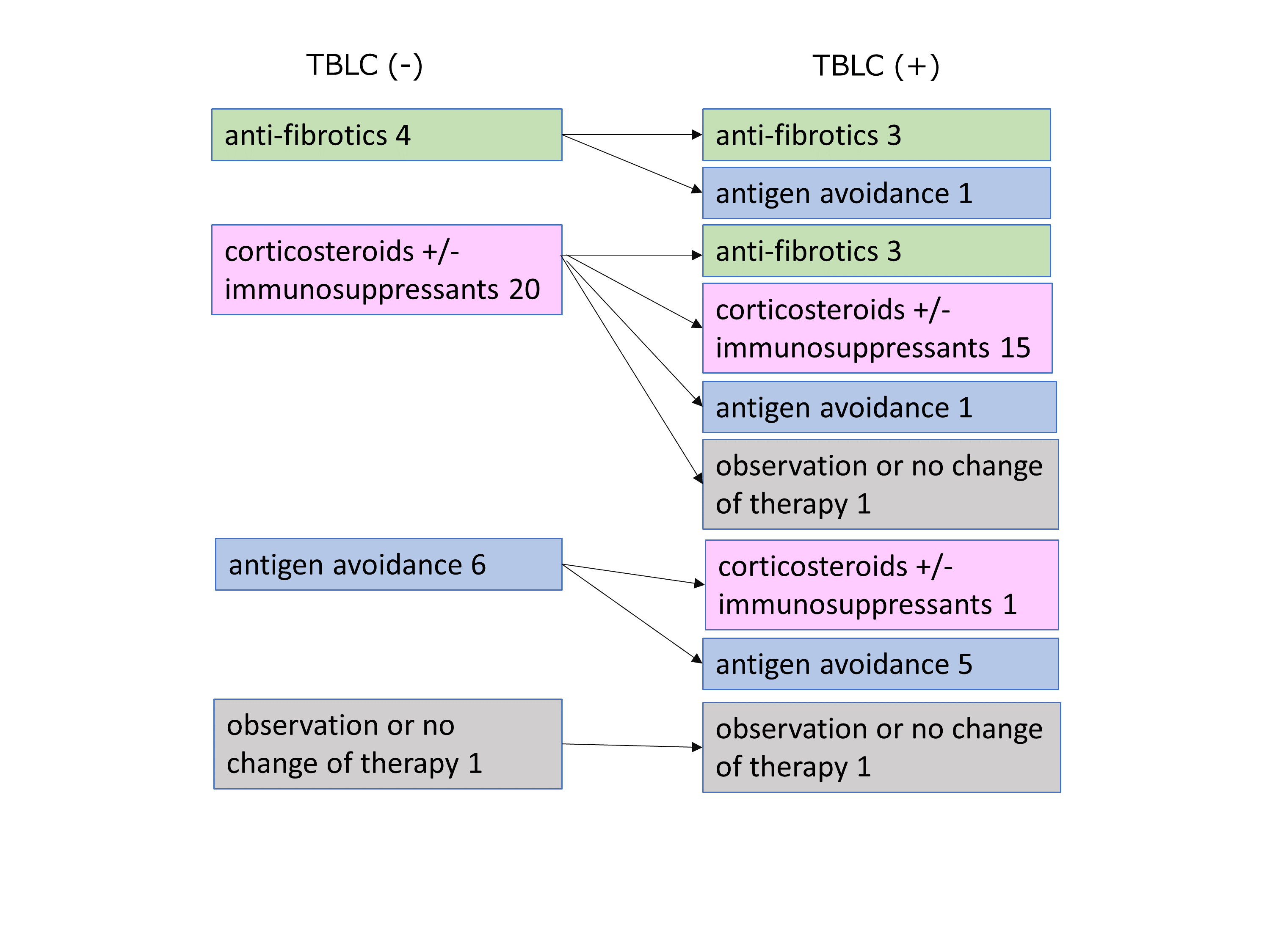

Supplement: Supplementary file 3 — Additional file 3: Fig. S3. Treatment strategy of pulmonologist A. Pulmonologist A selected treatment strategy based on clinical and radiological information without pathological information of TBLC and with pathological information of TBLC. The change of treatment strategy by TBLC was 7/31 (22.6%). [file 12890_2022_1838_MOESM3_ESM.png]

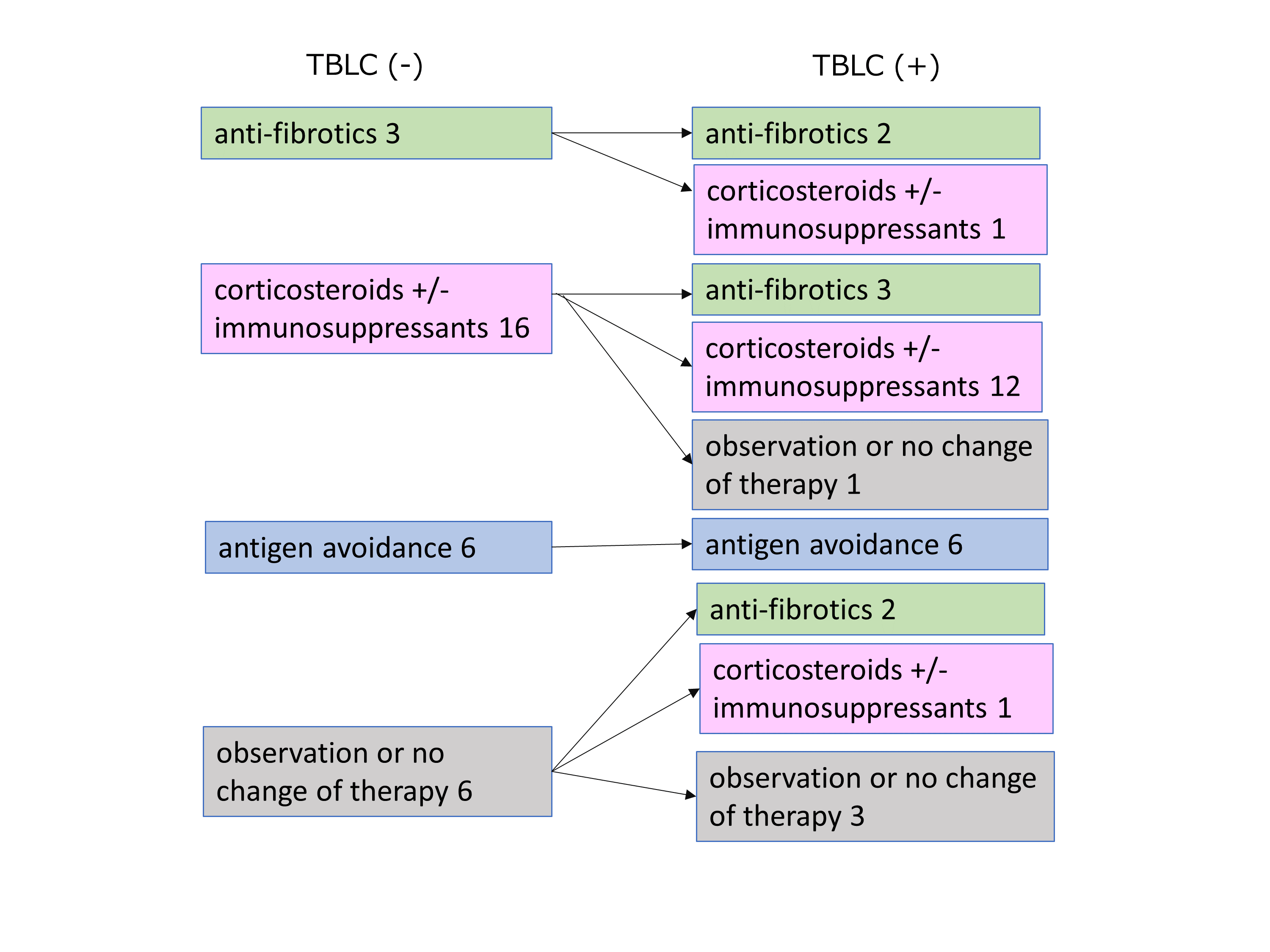

Supplement: Supplementary file 4 — Additional file 4: Fig. S4. Treatment strategy of pulmonologist B. Pulmonologist B selected treatment strategy based on clinical and radiological information without pathological information of TBLC and with pathological information of TBLC. The change of treatment strategy by TBLC was 8/31 (25.9%). [file 12890_2022_1838_MOESM4_ESM.png]

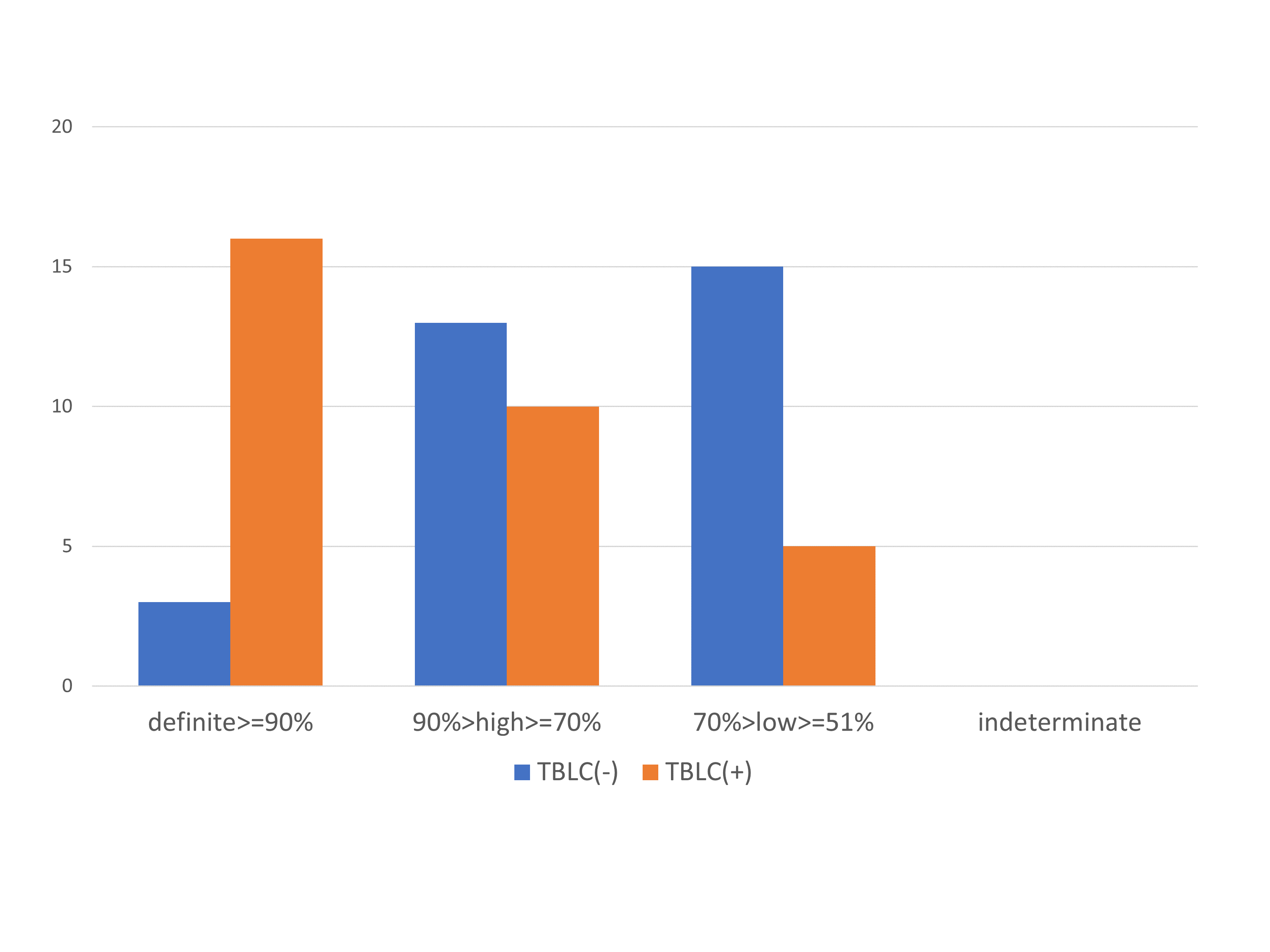

Supplement: Supplementary file 5 — Additional file 5: Fig. S5. Confidence level of pulmonologist A treatment strategy. Pulmonologist A selected confidence level of treatment strategy based on clinical and radiological information without pathological information of TBLC and with pathological information of TBLC. The definite or high confidence level of treatment strategy was 51.6% (16/31) without TBLC and 83.9% (26/31) with TBLC. [file 12890_2022_1838_MOESM5_ESM.png]

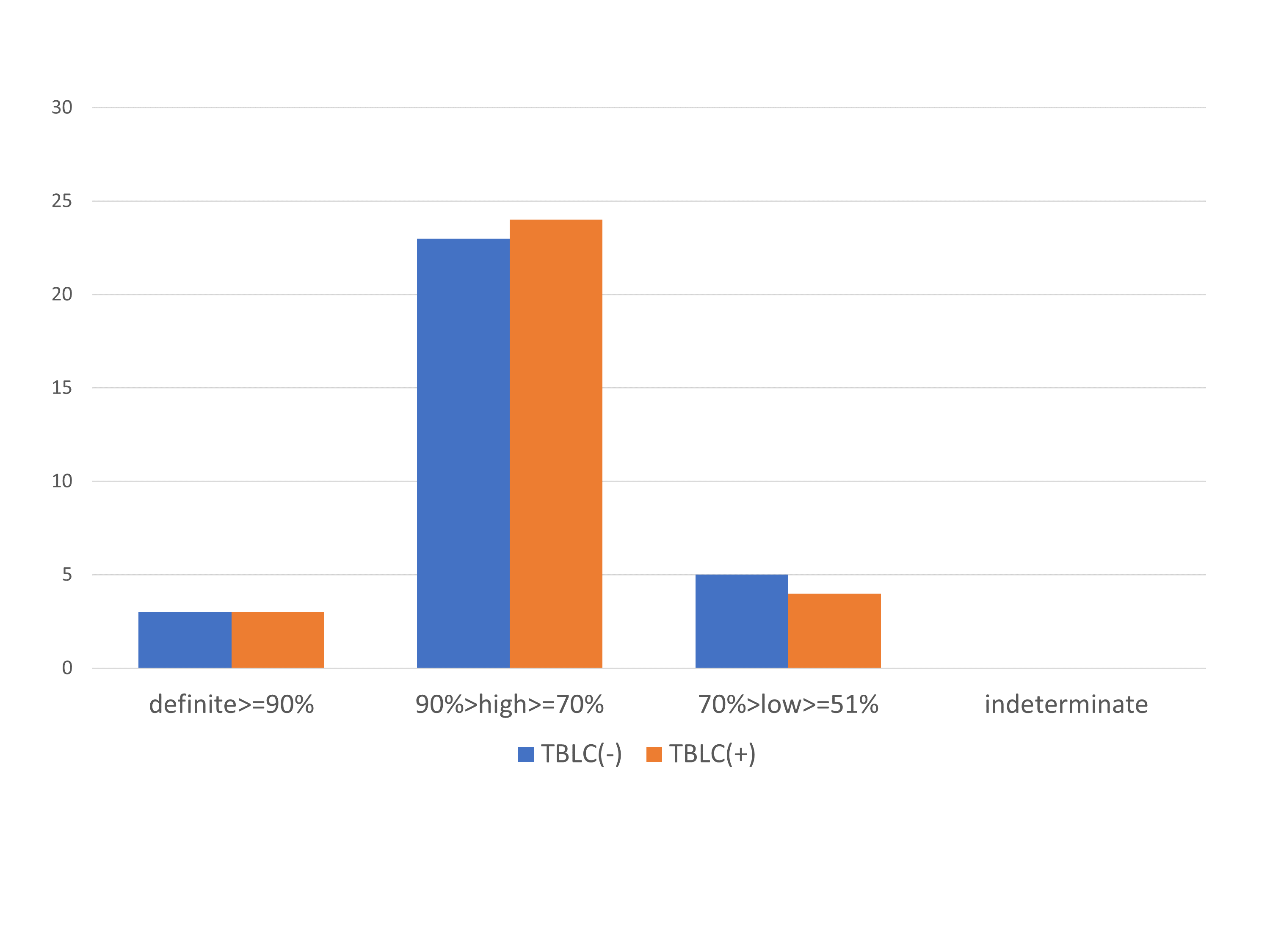

Supplement: Supplementary file 6 — Additional file 6: Fig. S6. Confidence level of pulmonologist B treatment strategy. Pulmonologist B selected confidence level of treatment strategy based on clinical and radiological information without pathological information of TBLC and with pathological information of TBLC. The definite or high confidence level of treatment strategy 83.9% (26/31) without TBLC and 87.1% (27/31) with TBLC. [file 12890_2022_1838_MOESM6_ESM.png]
